# Supplementary material for: Theoretical Analysis and Expression Profiling of 17β-Hydroxysteroid Dehydrogenase Genes in Gonadal Development and Steroidogenesis of Leopard Coral Grouper (Plectropomus leopardus)
Source: Int J Mol Sci. 2024 Feb 11;25(4):2180. doi: 10.3390/ijms25042180 (PMC10889806; doi:10.3390/ijms25042180)
Supplement: Supplementary file 1 [file ijms-25-02180-s001.zip › ijms-2812913-supplementary.pdf]

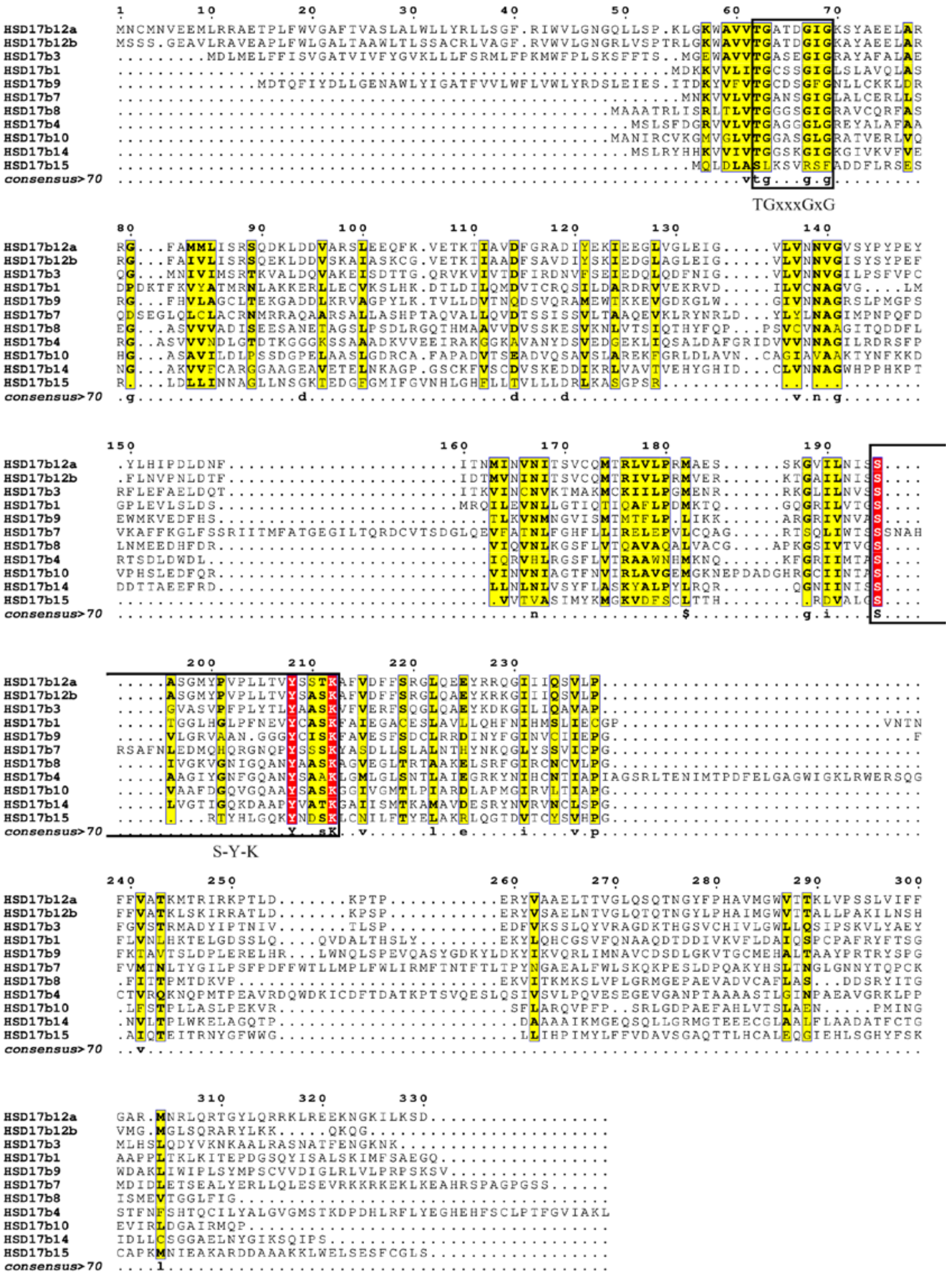

Figure S1. Amino Acid Alignment of the Hsd17b Gene Family in *P. leopardus*.
Note: Red indicates high sequence similarity, yellow indicates medium similarity.

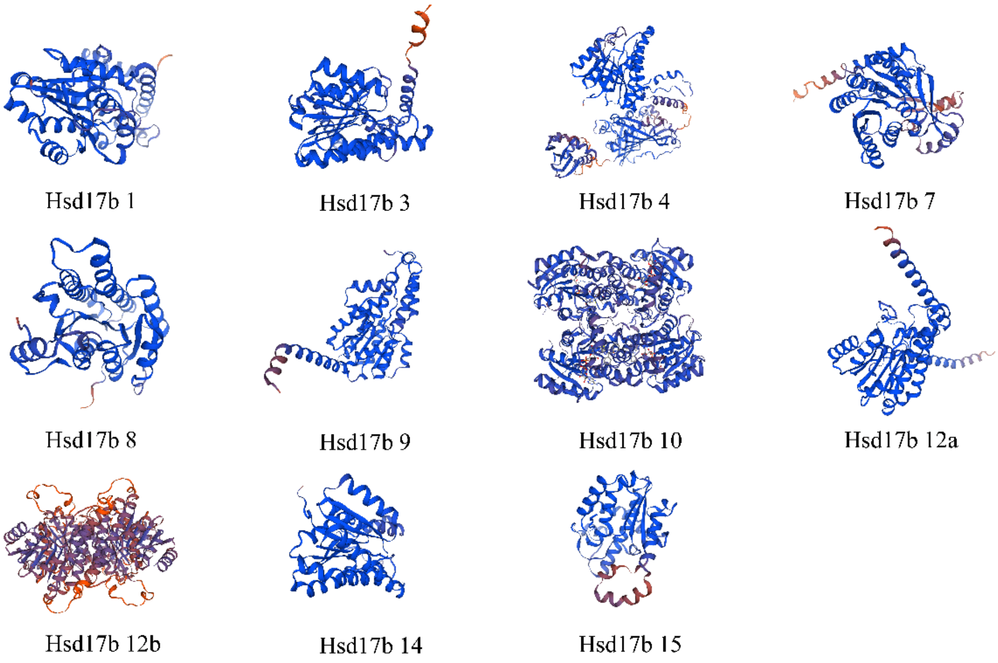

Figure S2. The protein structure of the flounder Hsd17b family genes in *P. leopardus*. The proteins modeling was made by SWISS-MODEL

Table S1. All species Hsd17b sequences used for phylogenetic tree construction.

Gene	Species	GenBank accession	Gene	Species	GenBank accession
hsd17b1	Homo sapiens	NP_000404.2	hsd17b8	Larimichthys crocea	XP_027142483.1
hsd17b1	*Mus musculus*	NP_034605.1	hsd17b8	Oreochromis niloticus	NP_001266465.1
hsd17b1	*Gallus gallus*	NP_990168.1	hsd17b8	Epinephelus lanceolatus	XP_033492268.1
hsd17b1	*Xenopus tropicalis*	XP_002935954.1	hsd17b8	Plectropomus leopardus	XP_042362813.1
hsd17b1	Danio rerio	NP_991147.2	hsd17b8	Cyprinus carpio	XP_042611619.1
hsd17b1	Paralichthys olivaceus	XP_019950004.1	hsd17b9	Homo sapiens	AAC50725.1
hsd17b1	Scophthalmus maximus	XP_035469907.1	hsd17b9	*Mus musculus*	NP_598767.1
hsd17b1	Lepisosteus oculatus	XP_015217570.1	hsd17b9	*Gallus gallus*	AAG00507.1
hsd17b1	Oryzias latipes	XP_004071345.1	hsd17b9	*Xenopus tropicalis*	XP_012812319.1
hsd17b1	Larimichthys crocea	XP_010737963.1	hsd17b9	Danio rerio	NP_001025272.1
hsd17b1	Oreochromis niloticus	XP_025761987.1	hsd17b9	Paralichthys olivaceus	XP_019952170.1
hsd17b1	Epinephelus lanceolatus	XP_033501630.1	hsd17b9	Scophthalmus maximus	XP_035488322.1
hsd17b1	Plectropomus leopardus	XP_042361394.1	hsd17b9	Lepisosteus oculatus	XP_006629411.1
hsd17b1	Cyprinus carpio	XP_042576382.1	hsd17b9	Oryzias latipes	XP_004068760.1
hsd17b2	Homo sapiens	NP_002144.1	hsd17b9	Larimichthys crocea	XP_027135011.1
hsd17b2	*Mus musculus*	NP_032316.2	hsd17b9	Oreochromis niloticus	XP_003456138.1
hsd17b2	*Gallus gallus*	XP_040537274.1	hsd17b9	Epinephelus lanceolatus	XP_033482037.1
hsd17b2	*Xenopus tropicalis*	XP_002934996.2	hsd17b9	Plectropomus leopardus	XP_042357765.1
hsd17b2	Danio rerio	NP_001035278.1	hsd17b10	Homo sapiens	NP_004484.1
hsd17b2	Scophthalmus maximus	XP_035497265.2	hsd17b10	*Mus musculus*	NP_058043.3
hsd17b2	Lepisosteus oculatus	XP_015223426.1	hsd17b10	*Gallus gallus*	XP_015128618.1
hsd17b2	Larimichthys crocea	XP_010753727.1	hsd17b10	*Xenopus tropicalis*	NP_001016511.1
hsd17b2	Epinephelus lanceolatus	XP_033491713.1	hsd17b10	Danio rerio	NP_001006098.1
hsd17b3	Homo sapiens	NP_000188.1	hsd17b10	Paralichthys olivaceus	XP_019967526.1
hsd17b3	*Mus musculus*	NP_032317.2	hsd17b10	Scophthalmus maximus	XP_035501223.1
hsd17b3	*Gallus gallus*	XP_425046.4	hsd17b10	Oryzias latipes	XP_004071072.1
hsd17b3	*Xenopus tropicalis*	XP_012827001.1	hsd17b10	Larimichthys crocea	XP_010740929.1
hsd17b3	Danio rerio	NP_956658.1	hsd17b10	Oreochromis niloticus	XP_003457109.1
hsd17b3	Paralichthys olivaceus	XP_019958261.1	hsd17b10	Epinephelus lanceolatus	XP_033484829.1
hsd17b3	Scophthalmus maximus	XP_035494692.1	hsd17b10	Plectropomus leopardus	XP_042347951.1
hsd17b3	Lepisosteus oculatus	XP_015224346.1	hsd17b10	Cyprinus carpio	XP_042568890.1
hsd17b3	Oryzias latipes	XP_004072273.2	hsd17b11	Homo sapiens	NP_057329.3
hsd17b3	Larimichthys crocea	XP_010731296.2	hsd17b11	*Mus musculus*	NP_444492.1
hsd17b3	Oreochromis niloticus	XP_003446190.1	hsd17b11	*Gallus gallus*	XP_040525634.1
hsd17b3	Epinephelus lanceolatus	XP_033476201.1	hsd17b12a	Homo sapiens	NP_057226.1
hsd17b3	Plectropomus leopardus	XP_042344332.1	hsd17b12a	*Mus musculus*	NP_062631.1
hsd17b4	Homo sapiens	NP_001186220.1	hsd17b12a	*Gallus gallus*	XP_015142684.1
hsd17b4	*Mus musculus*	NP_032318.2	hsd17b12a	*Xenopus tropicalis*	NP_001017234.1
hsd17b4	*Gallus gallus*	NP_990274.1	hsd17b12a	Danio rerio	XP_021332236.1
hsd17b4	*Xenopus tropicalis*	NP_001027490.1	hsd17b12a	Paralichthys olivaceus	XP_019934956.1

hsd17b4	Danio rerio	NP_956430.1	hsd17b12a	Scophthalmus maximus	XP_035499246.1
hsd17b4	Paralichthys olivaceus	XP_019954878.1	hsd17b12a	Lepisosteus oculatus	XP_006642505.1
hsd17b4	Scophthalmus maximus	XP_035494587.2	hsd17b12a	Oryzias latipes	XP_004069451.1
hsd17b4	Lepisosteus oculatus	XP_015222853.1	hsd17b12a	Larimichthys crocea	XP_027143858.1
hsd17b4	Oryzias latipes	XP_023814070.1	hsd17b12a	Oreochromis niloticus	NP_001266727.1
hsd17b4	Larimichthys crocea	XP_010731495.3	hsd17b12a	Epinephelus lanceolatus	XP_033490786.1
hsd17b4	Oreochromis niloticus	XP_003451110.1	hsd17b12a	Plectropomus leopardus	XP_042352113.1
hsd17b4	Epinephelus lanceolatus	XP_033475201.1	hsd17b12a	Cyprinus carpio	XP_042608972.1
hsd17b4	Plectropomus leopardus	XP_042344065.1	hsd17b12b	Danio rerio	NP_955907.1
hsd17b4	Cyprinus carpio	XP_042585087.1	hsd17b12b	Paralichthys olivaceus	XP_019954998.1
hsd17b5	Homo sapiens	NP_001240837.1	hsd17b12b	Scophthalmus maximus	XP_035484405.1
hsd17b5	*Mus musculus*	NP_085114.1	hsd17b12b	Oryzias latipes	XP_011485867.1
hsd17b6	Homo sapiens	NP_003716.2	hsd17b12b	Larimichthys crocea	XP_027137350.1
hsd17b6	*Mus musculus*	NP_038814.1	hsd17b12b	Oreochromis niloticus	XP_003450847.2
hsd17b7	Homo sapiens	NP_057455.1	hsd17b12b	Epinephelus lanceolatus	XP_033477993.1
hsd17b7	*Mus musculus*	NP_034606.3	hsd17b12b	Plectropomus leopardus	XP_042346655.1
hsd17b7	*Gallus gallus*	NP_001264435.1	hsd17b13	Homo sapiens	NP_835236.2
hsd17b7	*Xenopus tropicalis*	XP_031755934.1	hsd17b13	*Mus musculus*	NP_001156958.1
hsd17b7	Danio rerio	NP_001070796.1	hsd17b13	*Gallus gallus*	XP_040525633.1
hsd17b7	Paralichthys olivaceus	XP_019936533.1	hsd17b14	Homo sapiens	NP_057330.2
hsd17b7	Scophthalmus maximus	XP_035491323.1	hsd17b14	*Mus musculus*	NP_079606.3
hsd17b7	Lepisosteus oculatus	XP_006634994.2	hsd17b14	*Xenopus tropicalis*	XP_002935043.1
hsd17b7	Oryzias latipes	XP_011472673.1	hsd17b14	Danio rerio	NP_001003521.1
hsd17b7	Larimichthys crocea	XP_027146397.1	hsd17b14	Paralichthys olivaceus	XP_019950102.1
hsd17b7	Oreochromis niloticus	XP_003439749.1	hsd17b14	Scophthalmus maximus	XP_047183973.1
hsd17b7	Epinephelus lanceolatus	XP_033476979.1	hsd17b14	Oryzias latipes	XP_004071213.1
hsd17b7	Plectropomus leopardus	XP_042339482.1	hsd17b14	Larimichthys crocea	XP_010744565.3
hsd17b7	Cyprinus carpio	XP_042582004.1	hsd17b14	Oreochromis niloticus	XP_003442566.1
hsd17b8	Homo sapiens	NP_055049.1	hsd17b14	Epinephelus lanceolatus	XP_033499738.1
hsd17b8	*Mus musculus*	NP_038571.2	hsd17b14	Plectropomus leopardus	XP_042360641.1
hsd17b8	*Xenopus tropicalis*	NP_001016671.1	hsd17b15	Homo sapiens	NP_057110.3
hsd17b8	Danio rerio	NP_001005292.2	hsd17b15	*Mus musculus*	NP_067532.2
hsd17b8	Paralichthys olivaceus	XP_019963159.1	hsd17b15	Paralichthys olivaceus	XP_019963127.1
hsd17b8	Scophthalmus maximus	XP_035477126.1	hsd17b15	Plectropomus leopardus	XP_042370756.1
hsd17b8	Oryzias latipes	XP_004074105.1			

Table S2. Sequences of primers used in the study.

**Purpose**	**5′ to 3′ Sequence**
Hsd17b4-ISH-Fw	ATTTAGGTGACACTATAGAAGAGCAGCTATGATG GATGGCGGT
Hsd17b4-ISH-Rv	TAATACGACTCACTATAGGGAGAAGGACATGTCTT GCAGCGAA
Hsd17b12a-ISH-Fw	ATTTAGGTGACACTATAGAAGAGATGACTCGCATC AGGAAGCC
Hsd1712a-ISH-Rv	TAATACGACTCACTATAGGGAGAATCAGAGCAGA GTTTGCCGT
Vasa-ISH-Fw	ATTTAGGTGACACTATAGCTGATTTCCTCGCCGCT T
Vasa-ISH-Rv	TAATACGACTCACTATAGGGTGGCTCTTCACACCG TTGTC

Table S3. Secondary Structure Prediction of the Hsd17b Gene Family in *P. leopardus*.

Protein	Sequence length	Alpha helix (Hh)	Extended strand (Ee)	Beta turn (Tt)	Random coil (Cc)
Hsd17b1	290	52.76%	15.52%	8.62%	23.10%
Hsd17b3	320	49.06%	16.88%	7.19%	26.88%
Hsd17b4	734	33.11%	20.71%	8.31%	37.87%
Hsd17b7	348	41.09%	15.23%	6.61%	37.07%
Hsd17b8	256	44.53%	21.48%	8.59%	25.39%
Hsd17b9	332	52.71%	13.86%	8.13%	25.30%
Hsd17b10	260	39.62%	18.85%	9.23%	32.31%
Hsd17b12a	330	50.00%	15.76%	6.97%	27.27%
Hsd17b12b	319	49.84%	17.24%	6.58%	26.33%
Hsd17b14	264	39.39%	19.70%	8.71%	32.20%
HSD17b15	228	47.81%	14.47%	8.33%	29.39%

Table S4 The protein information of the Hsd17b Gene Family in *P. leopardus*.

Protein	Molecular weight (Da)	Theoretical pI	Grand average of Hydropathicity (GRAVY)	Instability index
Hsd17b1	31666.66	5.8	0.096	37.57
Hsd17b3	35609.66	8.89	0.251	44.58
Hsd17b4	79034.15	6.97	-0.116	33.25
Hsd17b7	38902.59	8.6	-0.15	38.84
Hsd17b8	26611.52	5.95	0.223	25.88
Hsd17b9	37294.38	8.47	0.031	30
Hsd17b10	27072.17	6.43	0.212	22.47
Hsd17b12a	37246.39	9.44	-0.002	43.96
Hsd17b12b	34674.46	9.7	0.216	38.16
Hsd17b14	28103.24	6.59	0.008	41.54
Hsd17b15	25391.26	7.67	0.027	40.49
